# Handwashing With a Water-Efficient Tap and Low-Cost Foaming Soap: The Povu Poa “Cool Foam” System in Kenya

**DOI:** 10.9745/GHSP-D-16-00022

**Published:** 2016-06-20

**Authors:** Jaynie Whinnery, Gauthami Penakalapati, Rachel Steinacher, Noel Wilson, Clair Null, Amy J Pickering

**Affiliations:** a Innovations for Poverty Action, Kenya, Kisumu, Kenya; b Catapult Design, San Francisco, CA, USA; c Stanford University, Stanford Center for Innovation in Global Health, Stanford, CA, USA

## Abstract

The new handwashing system, designed with end user input, features an economical foaming soap dispenser and a hygienic, water-efficient tap for use in household and institutional settings that lack reliable access to piped water. Cost of the soap and water needed for use is less than US$0.10 per 100 handwash uses, compared with US$0.20–$0.44 for conventional handwashing stations used in Kenya.

Using an interactive and iterative design approach involving representative end users, we created a new handwashing system in Kisumu, Kenya, to make handwashing convenient and economical in areas without reliable piped water. The innovative and adaptable system, branded as Povu Poa (“Cool Foam” in Kiswahili), integrates a cost-effective foaming soap dispenser with a hygienic, water-frugal water tap in a secure and affordable design.

## BACKGROUND

Handwashing with soap and water reduces the spread of respiratory and diarrheal disease, the 2 leading causes of death in children under 5 years old.^1^^-^^5^ Studies estimate that handwashing with soap can reduce acute respiratory infections by 21% and the risk of diarrhea by 40%.^6^^,^^7^

In settings without piped water, refilling water containers and securing soap for handwashing requires constant user effort and expense, creating barriers to handwashing with soap. In Kenya, for example, 78% of the population lacks access to household piped water,^8^ and the prevalence of handwashing with soap after contact with feces is estimated to be 15%.^6^

78% of the population in Kenya lacks access to household piped water, creating barriers to handwashing with soap.

**FIGURE 1:**
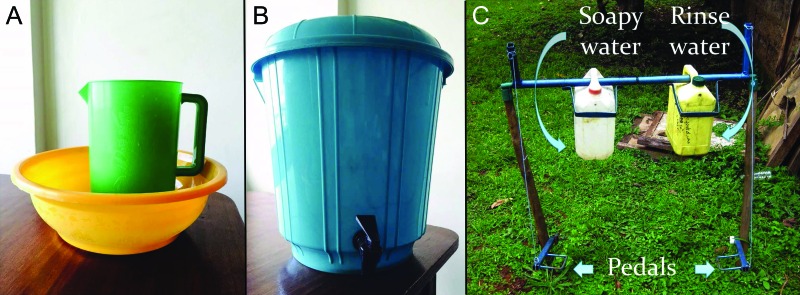
Conventional Handwashing Stations in Kenya

People are more likely to wash their hands at critical times if they have a dedicated place with soap and water.^9^ Conventional handwashing stations in Kenya, such as a jug and basin (Figure 1A) or a bucket with a tap (Figure 1B), are prone to soap theft, are cumbersome and unhygienic, and are not water-efficient. Alternative handwashing systems aim to provide affordable, water-efficient, and dedicated locations for handwashing. For example, the “leaky tin” dispenses water from a hole near the base of a container when a person removes a plug, and the “tippy tap” dispenses water by tipping the container when a person pulls on the attached string lever or steps on a foot pedal. However, difficulties with soap provision and security remain. The dual tippy tap integrates separate containers for soapy water and rinse water into a single system to address these issues (Figure 1C).^10^ The soapy water mixture, a 50:1 water-to-powdered soap ratio, increases the lifetime of the soap and is an effective cleansing agent.^11^ Still, the dual tippy tap has several shortcomings: it can become unstable over time, it requires frequent maintenance, the metal components are prone to theft, and the hardware is not particularly attractive.

## INNOVATION PROCESS

We began our design process by conducting in-depth interviews and focus group discussions with potential users in low-income, peri-urban areas of Kisumu, including household members in 5 households, students and teachers in 3 primary schools, and health care workers in 2 clinics. Users preferred hand washing systems that were easy to operate and refill with water, a tap that allowed them to control the flow of water, and a portable unit that they could store inside their home or institutions at night to prevent theft. We then created a series of handwashing system prototypes in response to user needs and iteratively developed the designs with multiple rounds of input from end users based on their experiences testing the various features.

After multiple rounds of user-focused testing with various handwashing components and systems, the final product we developed was a desirable, robust, affordable, and water-frugal system that integrates a secure soap dispenser with rinse water. We developed 2 configurations of the system, both of which are currently marketed under the brand Povu Poa (“Cool Foam” in Swahili).

**FIGURE 2:**
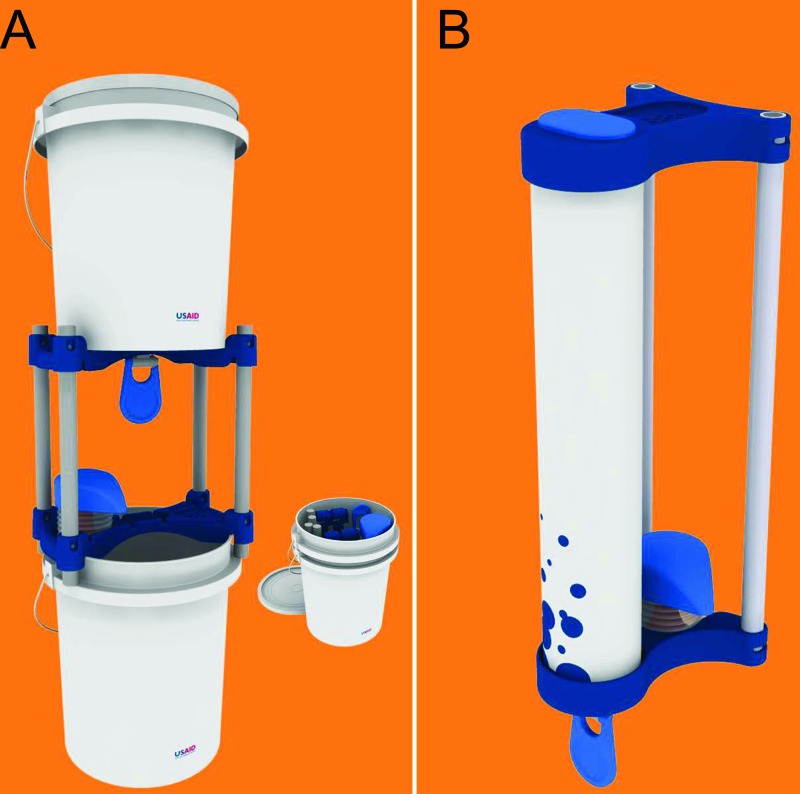
Configurations of the Povu Poa Handwashing System (A) bucket model design; (B) pipe model design.

- The Povu Poa **bucket model** is composed of two 20-liter buckets stacked vertically, which can be set on any level surface and easily disassembled for transport and security (Figure 2A).
- The Povu Poa **pipe model** is a light, highly portable 5-liter pipe that can be hung from a wall, fencepost, tree, or other standing structure and that can be plumbed to larger water tanks and drainage systems (Figure 2B).

The handwashing system we developed, marketed under the brand Povu Poa, comes in 2 configurations: a bucket and a pipe model.

**FIGURE 3:**
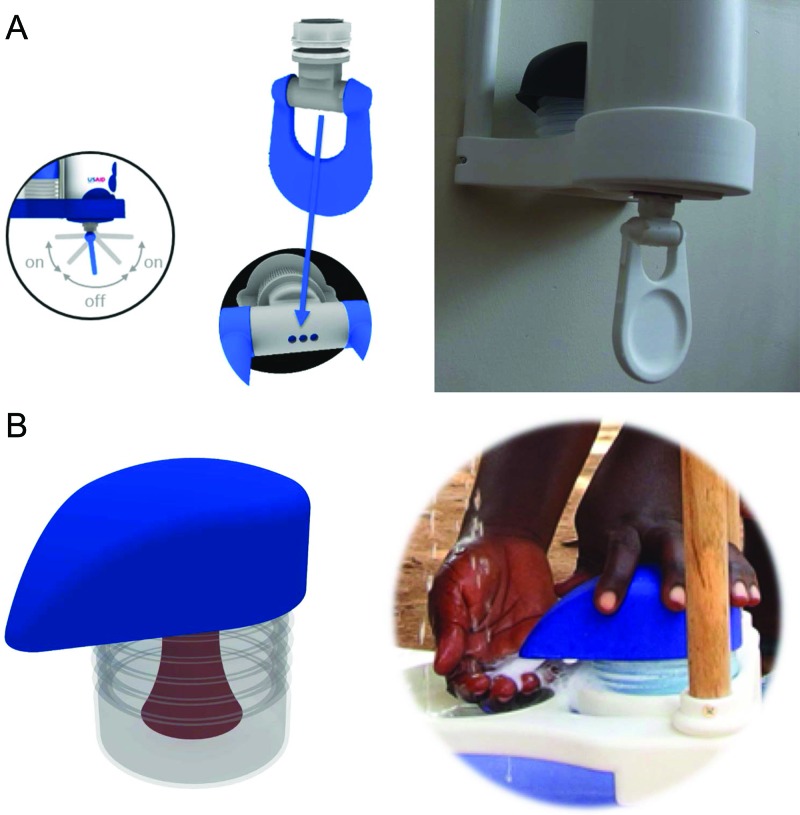
Water- and Soap-Dispensing Elements of the Povu Poa Handwashing System (A) Users can operate the Povu Poa Swing Tap hygienically with the back of their hand. The swing tap dispenses water from up to 3 holes; users can control the amount and flow of water coming from these holes based on how far forward or backward they pull/push the tap. (B) The Povu Poa Soap Foamer creates foam by mixing soapy water and air.

Both Povu Poa models integrate the water-frugal swing tap to dispense water (Figure 3A) and the accordion soap foamer that mixes soapy water with air to create a foam (Figure 3B). Runoff water from handwashing collects in the lower bucket for the bucket model or a separate basin for the pipe model (not shown).

## KEY PRODUCT FEATURES OF THE POVU POA HANDWASHING SYSTEM

**FIGURE 4:**
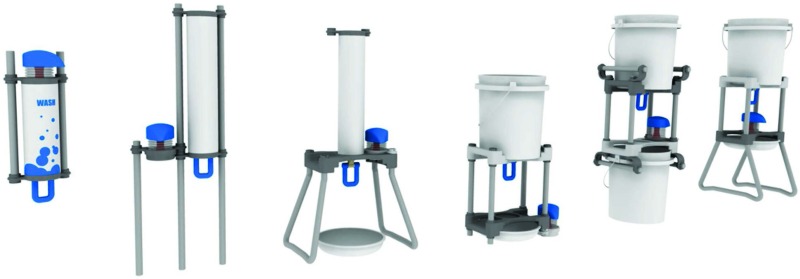
Potential Adaptations to the Povu Poa Handwashing System

- **Soap security:** The soap foamer is attached to the system, preventing theft.
- **Affordability:** Just 5 g of powdered or liquid soap mixed with 250 mL of water can provide 100 uses for US$0.10 (cost includes soap and water).
- **Hygienic:** The innovative swing-tap design is bidirectional and can be used with the back of the hand or wrist, limiting recontamination of hands after handwashing.
- **Water-frugality:** The water flow is sufficient for handwashing while providing a 30-77% reduction in water usage compared with conventional methods.
- **Scalability:** Components are specifically designed for low-cost mass production and deployment, estimated at US$12 per unit.
- **Adaptable:** The 2 handwashing station configurations can be adapted to meet different needs and preferences (Figure 4) and can be used in households and institutional settings, such as schools and health centers.

## SOAP AND WATER EFFICIENCY TESTING

We tested the water and the soap efficiency of the Povu Poa pipe and bucket prototypes alongside 6 handwashing systems commonly used in Kenya, such as the dual tippy tap and a jug and basin. At the beginning of each test, the system being evaluated was filled to capacity with water. For each test, research assistants from Innovations for Poverty Action washed their hands with soap and water for 20 seconds. Handwashing events continued intermittently until the water reservoir was empty. The total volume of water and handwash count were used to calculate water quantity per use. Before and after weights of the soap were used to calculate the amount of soap per use. Actual soap costs were used along with user-provided water prices.

The Povu Poa systems used 30% to 77% less water compared with the conventional systems tested, providing approximately 14 to 15 uses per 5 liters of water compared with 4 to 10 uses from the other systems (Table). The Povu Poa systems also used 94% to 99% less soap than the other tested systems, providing approximately 15,000 uses per US$1 spent on soap compared with approximately 500 to 1,600 uses with conventional systems. Overall the cost for soap and water with the Povu Poa is less than US$0.10 per 100 uses, compared with US$0.20 to US$0.44 per 100 uses for other systems tested. The water-frugal tap provides approximately 60 and 14 uses between refills for the bucket and pipe model, respectively.

The Povu Poa system uses 30%–77% less water than conventional handwashing stations used in Kenya.

**TABLE:** Water and Soap Efficiency for the Povu Poa Handwashing Prototypes Compared With Other Handwashing Devices

	Soap Type	Water Quantity per Use (mL)	No. of Uses per 5 L of Water	No. of Uses per US$1 Spent on Water	Amount of Soap per Use (g)	No. of Uses per US$1 Spent on Soap	Cost in US$ for Soap and Water per 100 Uses
Povu Poa pipe model	Soapy water made with Omo brand powdered soap	357	14	1,064	0.03	14,865	$0.10
Povu Poa bucket model	Soapy water made with Omo brand powdered soap	333	15	1,140	0.03	15,696	$0.09
Dual tippy tap	Soapy water made with Omo brand powdered soap	625	8	608	0.96	500	$0.37
Jug and basin	Multipurpose bar soap (Toyo brand)	513	10	741	0.49	1,600	$0.20
Sink with metal tap	Multipurpose bar soap (Toyo brand)	1,429	4	266	0.71	1,100	$0.47
20 L barrel with metal tap	Locally made liquid soap	690	7	551	1.90	800	$0.30
20 L barrel with plastic tap	Locally made liquid soap	1,000	5	380	2.85	550	$0.44
15 L bucket with plastic tap	Multipurpose bar soap (Toyo brand)	833	6	456	0.89	900	$0.33

Based on these results and our estimated mass production cost of US$12 for the Povu Poa pipe model, the pipe model would pay for itself in approximately 2.5 years for a family of 5 who each wash their hands 3 times per day using a jug and basin. When considering the soap foamer alone, at a mass production price of US$3, the soap foamer would pay for itself in just 1 year using the same assumptions and the calculated cost savings of soap.

The Povu Poa pipe model would pay for itself in about 2.5 years for a family of 5.

## CURRENT AND FUTURE WORK

In focus group discussions, approximately 80% of participants stated they would purchase a Povu Poa product, suggesting the aspirational value of the product. We have produced 200 Povu Poa systems in Kenya and are currently field testing them in peri-urban households, schools, and health clinics to assess long-term usage (up to 1 year of evaluation) and durability. To assess demand for the product, Povu Poa units are currently being sold to households at randomized price points, ranging from US$1 to US$12, to determine the price that most low-income users are willing and able to pay. Next steps include finalizing the design for mass production of the Povu Poa system, partnering with a plastics manufacturer, and identifying effective sales and distribution strategies.

We have produced 200 Povu Poa systems in Kenya and are currently field testing them in several locations.

## Notes

**Peer Reviewed**

## References

[1] Bhutta ZA Das JK Walker N Rizvi A Campbell H Rudan I Interventions to address deaths from childhood pneumonia and diarrhoea equitably: what works and at what cost? Lancet. 2013;381(9875):1417–1429. 10.1016/S0140-6736(13)60648-0. 23582723

[2] Bowen A Agboatwalla M Luby S Tobery T Ayers T Hoekstra RM. Association between intensive handwashing promotion and child development in Karachi, Pakistan: a cluster randomized controlled trial. Arch Pediatr Adolesc Med. 2012;166(11):1037–1044. 10.1001/archpediatrics.2012.1181. 22986783 PMC4648282

[3] Curtis V Cairncross S. Effect of washing hands with soap on diarrhoea risk in the community: a systematic review. Lancet Infect Dis. 2003;3(5):275–281. 10.1016/S1473-3099(03)00606-6. 12726975

[4] Rabie T Curtis V. Handwashing and risk of respiratory infections: a quantitative systematic review. Trop Med Int Health. 2006;11(3):258–267. 10.1111/j.1365-3156.2006.01568.x. 16553905 PMC7169664

[5] Walker CL Rudan I Liu L Nair H Theodoratou E Bhutta ZA Global burden of childhood pneumonia and diarrhoea. Lancet. 2013;381(9875):1405–1416. 10.1016/S0140-6736(13)60222-6. 23582727 PMC7159282

[6] Freeman MC Stocks ME Cumming O Jeandron A Higgins JP Wolf J Hygiene and health: systematic review of handwashing practices worldwide and update of health effects. Trop Med Int Health. 2014;19(8):906–916. 10.1111/tmi.12339. 24889816

[7] Aiello AE Coulborn RM Perez V Larson EL. Effect of hand hygiene on infectious disease risk in the community setting: a meta-analysis. Am J Public Health. 2008;98(8):1372–1381. 10.2105/AJPH.2007.124610. 18556606 PMC2446461

[8] United Nations Children’s Fund (UNICEF); World Health Organization (WHO). Progress on sanitation and drinking water: 2015 update and MDG assessment. New York: UNICEF; 2015 Co-published by WHO. Available from: http://www.unicef.org/publications/files/Progress_on_Sanitation_and_Drinking_Water_2015_Update_.pdf

[9] Luby SP Haldexsr AK Tronchet C Akhter S Bhuiya A Johnston RB. Household characteristics associated with handwashing with soap in rural Bangladesh. Am J Trop Med Hyg. 2009;81(5):882–887. 10.4269/ajtmh.2009.09-0031. 19861626

[10] Arnold BF Null C Luby SP Unicomb L Stewart CP Dewey KG Cluster-randomised controlled trials of individual and combined water, sanitation, hygiene and nutritional interventions in rural Bangladesh and Kenya: the WASH Benefits study design and rationale. BMJ Open. 2013;3(8):e003476. 10.1136/bmjopen-2013-003476. 23996605 PMC3758977

[11] Amin N Pickering AJ Ram PK Unicomb L Najnin N Homaira N Microbiological evaluation of the efficacy of soapy water to clean hands: a randomized, non-inferiority field trial. Am J Trop Med Hyg. 2014;91(2):415–423. 10.4269/ajtmh.13-0475. 24914003 PMC4125272

